# Assessing Women’s Knowledge and Awareness of Sexually Transmitted Infections in Saudi Arabia: A Comprehensive Study

**DOI:** 10.3390/healthcare12141437

**Published:** 2024-07-18

**Authors:** Nujud Hassan Al-sahli, Zahra Essa Alhammaqi, Raghad Faisal Alruwailiy, Shatha Ahmed Alzahrani, Asma Ahmed Hakami, Ashjan Saeed Al Mansour, Ola Abdu Yahya Khawaji, Hanadi Bakhsh

**Affiliations:** 1 Collage of Nursing, Princess Nora Bint Abdul Rahman University, Riyadh 11564, Saudi Arabia; hadh4008@gmail.com; 2 Faculty of Medical Sciences, Medicine, University of Groningen, 9712 CP Groningen, The Netherlands; 3 Collage of Medicine, Northern Border University, Arar 73213, Saudi Arabia; st201903099@stu.nbu.edu.sa; 4 Collage of Nursing, Al Baha University, Al-Baha 65779, Saudi Arabia; 441008362@stu.bu.edu.sa; 5 College of Medicine, Jazan University, Jazan 45142, Saudi Arabia; 202203728@stu.jazanu.edu.sa (A.A.H.); 201905940@stu.jazanu.edu.sa (O.A.Y.K.); 6 College of Medicine, Najran University, Najran 66462, Saudi Arabia; 7 Obstetrics and Gynecology Department, College of Medicine, Princess Nourah Bint Abdulrahman University, Riyadh 11564, Saudi Arabia

**Keywords:** sexually transmitted infections, knowledge, awareness, misconceptions, Saudi Arabia

## Abstract

This study aimed to comprehensively assess the knowledge, awareness, and misconceptions regarding sexually transmitted infections (STIs) among women in Saudi Arabia. A cross-sectional survey was conducted with 600 women aged 18–55 from various regions across the country. The findings revealed moderate overall STI knowledge, with gaps in understanding transmission routes (31.7%), recognizing symptoms (40.8%), and awareness of prevention methods (35.2%). Prevalent misconceptions included the belief that STIs can spread through casual contact (38%), only individuals with multiple partners are at risk (30%), and STIs are always symptomatic (32%). Demographic factors such as age, education level, and marital status significantly influenced STI knowledge, while residential area did not. Higher education, particularly bachelor’s degrees and above, was strongly associated with better awareness. Digital platforms like the internet and social media emerged as significant sources of STI information. Undergoing STI testing, discussing STIs with partners, using protection, and receiving the HPV vaccine were linked to higher knowledge levels. This study highlights the need for targeted educational interventions, integration of sexual health education into curricula, training healthcare providers, community engagement, and leveraging digital platforms to enhance STI awareness and prevention efforts among Saudi women.

## 1. Introduction

Sexually transmitted infections (STIs) represent a significant public health challenge globally, affecting millions of individuals each year [1]. The World Health Organization (WHO) reports that over one million new cases of STIs occur daily worldwide. These infections can lead to severe health complications, including infertility, ectopic pregnancies, chronic pelvic pain, and increased susceptibility to HIV [2,3]. Despite their prevalence and impact, STIs are often surrounded by stigma, misinformation, and a lack of awareness, particularly in regions with conservative cultural norms [4]. This is particularly relevant in Saudi Arabia, where traditional cultural values and religious beliefs significantly influence sexual health education and awareness [5,6].

In Saudi Arabia, the social and cultural context presents unique challenges for STI awareness and prevention. The country’s conservative stance on issues related to sexuality and sexual health can hinder open discussion and education on STIs [7,8]. This environment contributes to a lack of comprehensive sexual health education, which is crucial for effective STI prevention and management [9,10]. Studies have shown that in societies where sexuality is a taboo subject, there is often a significant gap in knowledge regarding STIs, their transmission, symptoms, and prevention methods. This knowledge gap can lead to delays in seeking treatment and contribute to the spread of infections [11,12].

Previous research in the Middle East has highlighted a general lack of awareness and misconceptions about STIs among the population. For instance, a study conducted in Oman revealed that many women lacked basic knowledge about common STIs, their symptoms, and prevention strategies [6,12,13]. Similarly, research in Saudi indicated that misconceptions and a lack of information were prevalent among both men and women regarding STIs. These findings underscore the need for culturally tailored educational programs to address the specific needs and contexts of different populations within the region [14,15].

In Saudi Arabia, few studies have comprehensively assessed women’s knowledge and awareness of STIs. One notable study conducted by Fageeh et al. (2022) found that among female university students in Riyadh, there was a moderate level of awareness about STIs, but significant gaps remained in understanding transmission routes and prevention methods [16]. Another study by Malli et al. (2023) highlighted that although some women were aware of the existence of STIs, many held misconceptions about their severity and prevention [14]. These studies suggest that while there is some level of awareness among educated women, there is a need for more comprehensive and accessible educational resources across different demographics [17,18].

The limited availability of sexual health education in Saudi Arabia is influenced by several factors. Cultural norms and religious beliefs play a critical role in shaping attitudes towards sexual health and education [19]. The education system in Saudi Arabia traditionally places little emphasis on sexual health, leading to significant gaps in knowledge among young people [20]. Moreover, public health campaigns and resources on STIs are not as visible or comprehensive compared to other regions due to cultural sensitivities surrounding the topic.

Access to healthcare and STI services also varies across different regions of Saudi Arabia [21]. Urban areas, such as Riyadh and Jeddah, have more advanced healthcare facilities and better access to STI testing and treatment services compared to rural areas [22]. Women in rural areas may face additional barriers, including limited access to healthcare facilities, lower levels of education, and cultural restrictions that may prevent them from seeking information and care for STIs. This disparity highlights the importance of addressing geographical and socioeconomic factors in STI awareness and prevention efforts [23].

Efforts to improve STI awareness and education in Saudi Arabia must consider the cultural and social context. Culturally appropriate education strategies are essential to effectively communicate information about STIs and reduce the stigma associated with them [24]. Initiatives such as incorporating STI education into school curricula, leveraging social media for public health campaigns, and training healthcare providers to discuss sexual health in a culturally sensitive manner can play a significant role in enhancing awareness [25]. Additionally, engaging community leaders and influencers in promoting sexual health can help overcome cultural barriers and facilitate more open discussions about STIs [26].

Internationally, there are various models of successful STI education and prevention programs that can be adapted to the Saudi context [27]. For example, comprehensive sex education programs in countries like the Netherlands and Sweden have been effective in reducing STI rates and increasing awareness among young people [28]. These programs typically include information on the biology of STIs, modes of transmission, prevention methods, and the importance of regular testing [29]. Adaptation of these models to the Saudi context would require careful consideration of cultural values and norms to ensure acceptance and effectiveness [30,31].

Digital platforms and mobile health (mHealth) initiatives also offer promising avenues for enhancing STI awareness and education in Saudi Arabia [32]. The widespread use of smartphones and the internet among the Saudi population presents an opportunity to reach a broad audience with educational content [33]. Mobile applications and online resources can provide discreet and accessible information about STIs, encourage regular testing, and connect users with healthcare services [34]. These digital tools can be particularly valuable in reaching young people and those who may feel uncomfortable discussing sexual health issues in person [35].

Despite these potential solutions, challenges remain in implementing effective STI education and prevention strategies in Saudi Arabia. The conservative social climate may limit the scope of public health campaigns and the integration of sexual health education into formal curricula [36]. Additionally, addressing the stigma associated with STIs requires a multifaceted approach that includes education, community engagement, and policy changes [37]. Overcoming these challenges will require collaboration between government agencies, healthcare providers, educators, and community leaders [38].

The issue of STIs in Saudi Arabia is compounded by a lack of awareness, cultural sensitivities, and limited access to comprehensive sexual health education [38]. Addressing these challenges requires culturally tailored educational initiatives, improved healthcare access, and the use of digital tools to reach a wider audience [39]. By enhancing STI awareness and knowledge among women, Saudi Arabia can make significant strides in reducing the prevalence of STIs and improving overall public health [40].

### 1.1. Aim of the Study

The aim of this study is to comprehensively assess the level of knowledge, awareness, and misconceptions about sexually transmitted infections (STIs) among women in Saudi Arabia. The study seeks to identify demographic factors influencing STI awareness and to evaluate the effectiveness of current educational resources and public health initiatives. By understanding these aspects, the study aims to inform the development of targeted educational interventions and public health strategies that are culturally appropriate and effective in improving STI awareness and prevention among women in Saudi Arabia.

### 1.2. Research Questions

1. What is the current level of knowledge and awareness about sexually transmitted infections among women in Saudi Arabia, and how does this vary across different demographic groups?
2. What are the common misconceptions about sexually transmitted infections among women in Saudi Arabia, and what are the perceived barriers to accessing accurate information and healthcare services related to STIs?

## 2. Materials and Methods

### 2.1. Study Design and Setting

This study utilized a cross-sectional design to evaluate the awareness and knowledge of sexually transmitted infections (STIs) among women in Saudi Arabia. Data were collected between November 2023 and January 2024 across the main regions of Saudi Arabia, including the Central, Eastern, Northern, Southern, and Western regions.

### 2.2. Participants and Sampling

#### 2.2.1. Target Population

The target population for this study comprised women aged 18 to 55 years residing in various regions across Saudi Arabia. This age range was selected to include women who are likely to be sexually active or approaching menopause, thereby encompassing a broad spectrum of potential experiences and knowledge levels regarding sexually transmitted infections (STIs). The regions covered include the Central, Eastern, Northern, Southern, and Western parts of Saudi Arabia, representing diverse geographical and socio-cultural backgrounds.

#### 2.2.2. Sampling Method

A stratified random sampling technique was employed to ensure a representative sample of the target population. This method involves dividing the population into distinct subgroups or strata based on key demographic variables, and then randomly selecting participants from each stratum. The strata were defined as follows:

1. Age Groups: 18–23, 24–30, 31–40, 41–50, and 51–55 years.
2. Education Levels: Non-formal Education, elementary school, intermediate school, secondary school, diploma, bachelor’s degree, master’s degree, and Ph.D.
3. Residential Areas: Central, Eastern, Northern, Southern, and Western regions.

Proportional allocation was used to ensure that the sample size from each stratum was proportional to its size in the overall population. This approach helped achieve a balanced representation, considering the differences in population density and demographics across the regions.

#### 2.2.3. Sample Size Calculation

The sample size was determined using a sample size calculator to ensure sufficient power for statistical analysis. Key parameters included the following:

- Confidence Level: 95%.
- Margin of Error: 4%.
- Estimated Prevalence: Assumed to be 50% to maximize the sample size.

Using these parameters, the calculated sample size was 600 participants. This number was chosen to ensure adequate representation and statistical significance, allowing for robust analysis of the data.

#### 2.2.4. Recruitment Process

Participants were recruited through an online survey distributed via various social media platforms, including WhatsApp, Twitter, Facebook, and Instagram. The survey link was shared in both Arabic and English to accommodate language preferences and increase reach. To encourage participation and ensure a diverse sample, the recruitment strategy included the following:

1. Broadcast Messages: Disseminating the survey link through popular social media groups and networks catering to different age groups and regions.
2. Influencer Collaboration: Engaging social media influencers and community leaders to promote the survey and encourage participation from their followers.

#### 2.2.5. Inclusion and Exclusion Criteria

- Inclusion Criteria:
  ○ Women aged 18–55 years.
  ○ Residents of Saudi Arabia.
  ○ Willing to provide informed consent and participate in the survey.
- Exclusion Criteria:
  ○ Women outside the specified age range.
  ○ Non-residents of Saudi Arabia.
  ○ Participants who did not consent to participate or withdrew consent during the study.

#### 2.2.6. Recruitment Process

Recruiting participants was indeed challenging, and we employed several techniques to ensure a diverse and representative sample. Our recruitment strategies included the following:

1. Social Media Engagement: We leveraged popular social media platforms such as WhatsApp, Twitter, Facebook, and Instagram to disseminate the survey link. Posts were crafted to be engaging and informative, emphasizing the importance of participation.
2. Influencer Collaboration: We collaborated with social media influencers and community leaders who promoted the survey link to their followers, thereby increasing our reach.
3. Email Invitations: We sent email invitations to potential participants, including students, professionals, and community members, to target demographics that might be underrepresented in social media outreach.
4. Broadcast Messages: We disseminated the survey link through popular social media groups and networks catering to different age groups and regions.

We did not provide any monetary incentives; however, we emphasized the importance of this study and its potential impact on public health policies and interventions. This sense of contributing to an important cause motivated many participants.

### 2.3. Data Collection

#### 2.3.1. Development of the Questionnaire

The questionnaire was meticulously developed based on existing literature and validated tools used in previous studies on STI awareness. It aimed to capture a comprehensive picture of women’s knowledge, awareness, misconceptions, and practices related to STIs in Saudi Arabia. The questionnaire was designed to be user-friendly and accessible, ensuring clarity and ease of understanding for participants from diverse educational backgrounds.

#### 2.3.2. Sections of the Questionnaire

1. Demographic Information: Age, marital status, educational level, and residential area.
2. STI Knowledge Assessment: Questions about the identification of common STIs (such as chlamydia, gonorrhea, syphilis, HPV, and herpes), modes of transmission, symptoms, prevention strategies, and sources of information. Knowledge questions were crafted to include multiple-choice and true/false formats to objectively assess participants’ understanding.
3. Misconceptions and Stigma: Items addressing common misconceptions about STIs, including myths and cultural beliefs that might influence understanding and attitudes towards STIs.
4. Personal Practices and Behaviors: Queries regarding participants’ history of STI testing, vaccination, discussions with partners about STIs, and use of protective measures during sexual intercourse.
5. Information Sources: Questions about where participants obtained their information on STIs, including healthcare providers, educational materials, media, and peer discussions.

The questionnaire was pre-tested with a small group of women representative of the target population to identify and rectify any ambiguities or misunderstandings. Adjustments were made based on feedback to enhance the reliability and validity of the questions.

#### 2.3.3. Distribution and Administration

Digital Platform: The final questionnaire was digitized using Google Forms, allowing for easy distribution and real-time data collection. The online format facilitated wide-reaching dissemination across diverse geographic locations in Saudi Arabia, minimizing logistical challenges associated with paper-based surveys.

Language and Accessibility: The questionnaire was provided in both Arabic and English to accommodate participants’ language preferences. Instructions and explanations were included to guide participants through the process, ensuring they understood the purpose of this study, the nature of the questions, and the confidentiality of their responses.

#### 2.3.4. Dissemination Strategy

1. Social Media Platforms: The questionnaire link was shared through popular social media platforms such as Twitter, Facebook, and Instagram, which are widely used in Saudi Arabia. Posts and messages were crafted to be engaging and informative, emphasizing the importance of participation in the study.
2. WhatsApp Groups: Recognizing the widespread use of WhatsApp in Saudi Arabia, the questionnaire link was also distributed through various WhatsApp groups, including those focused on women’s health, educational communities, and local social circles.
3. Email Invitations: Invitations to participate in this study were sent via email to potential participants, including students, professionals, and community members. This approach targeted specific demographics that might be underrepresented in social media outreach.

Informed Consent: Before accessing the questionnaire, participants were required to read a consent statement outlining the study’s objectives, the voluntary nature of participation, and assurances of confidentiality. Participants had to explicitly agree to these terms by selecting an “I Consent” option before proceeding to the survey questions.

#### 2.3.5. Data Collection Period

Data collection occurred over a three-month period from November 2023 to January 2024. This timeline allowed for sufficient data accumulation while ensuring the study remained current and reflective of recent awareness and behaviors regarding STIs.

#### 2.3.6. Ethical Considerations

This study adhered to stringent ethical standards to ensure the protection of participants’ rights and the integrity of the research process. Ethical approval was obtained from the Princess Nourah University Institutional Review Board (IRB: 23-0790) before the commencement of the study, ensuring that all procedures complied with institutional guidelines and international ethical norms. Participants were provided with a clear and comprehensive informed consent statement before participation, detailing the study’s objectives, the voluntary nature of their involvement, and their right to withdraw at any time without any adverse consequences. Participants were also informed of their right to contact the research team with any questions or concerns, promoting transparency and fostering trust. This rigorous ethical approach was integral to upholding participants’ rights, maintaining data integrity, and ensuring the overall credibility of the study.

#### 2.3.7. Statistical Analysis

Statistical analysis was conducted using IBM SPSS Statistics version 27.0.1. Descriptive statistics summarized demographic variables, such as age, education level, marital status, and residential area, as frequencies and percentages. To evaluate associations between these demographics and levels of STI knowledge, a Chi-square test was applied. Significant relationships were determined with a *p*-value threshold of <0.05.

Logistic regression analysis further examined the influence of demographic factors on STI knowledge, providing odds ratios (ORs) and 95% confidence intervals (CIs) to identify significant predictors while controlling for potential confounders. This multivariate approach allowed us to quantify how specific demographics impacted the likelihood of higher STI knowledge levels, contributing to a comprehensive understanding of awareness variations among the participants.

## 3. Results

This study included a diverse sample of 600 women aged 18–55 from various regions in Saudi Arabia (Table 1). The majority of participants were in the 24–30 (25.3%) and 31–40 (23.5%) age groups, with the smallest group being 51–55 years (13.5%). Educational levels varied, with the largest proportion holding a bachelor’s degree (31.3%), followed by secondary school education (17.2%) and diplomas (14.5%). Illiteracy was relatively low at 4.7%. The residential distribution showed a balanced representation from different regions, with the Western region (25.2%) having the highest representation. Regarding marital status, a majority were married (46.3%), followed by single (34.8%), divorced (12.2%), and widowed participants (6.7%). These demographics provide a comprehensive view of the study population, allowing for a detailed analysis of STI awareness across different age, education, and regional groups.

Table 2 illustrates a varied level of knowledge regarding sexually transmitted infections (STIs) among women in Saudi Arabia. A significant 72.5% of participants were able to identify common STIs, indicating a relatively high level of awareness; however, 27.5% were not familiar with these infections. Understanding of STI transmission routes was evident in 68.3% of respondents, suggesting a fairly good comprehension of how STIs spread, though 31.7% lacked this crucial knowledge. Knowledge of STI symptoms was notably lower, with only 59.2% correctly identifying symptoms, leaving 40.8% either unaware or misinformed. Awareness of STI prevention methods was present in 64.8% of the participants, indicating a reasonable understanding of protective measures, but 35.2% did not know effective prevention strategies.

The data presented in Table 3 reveal significant misconceptions about sexually transmitted infections (STIs) among women in Saudi Arabia. Notably, 38% of participants believe that STIs can be transmitted through casual contact, while 49% correctly disagree with this statement, and 13% are unsure. Additionally, 30% of respondents incorrectly believe that only individuals with multiple sexual partners are at risk of contracting STIs, with 56% disagreeing and 14% uncertain.

The belief that STIs are always visible with symptoms is held by 32% of participants, suggesting a lack of awareness about asymptomatic cases, while 52% disagree, and 16% are unsure. This misunderstanding could lead to delays in seeking treatment and contribute to the spread of infections. Finally, 40% of respondents believe that condoms completely eliminate the risk of STIs, indicating overconfidence in this prevention method, despite 48% correctly recognizing that condoms reduce but do not entirely eliminate the risk, and 12% are unsure.

The data in Table 4 reveal diverse sources of information regarding sexually transmitted infections (STIs) among women in Saudi Arabia. The most commonly cited source of information is the internet/websites, with 260 participants (43.3%) indicating that they rely on online resources. This is closely followed by social media, which serves as a significant information source for 245 women (40.8%), reflecting the growing influence of digital platforms on health information dissemination.

Healthcare providers are the primary source for 215 participants (35.8%), underscoring the crucial role of medical professionals in STI education. However, only 105 women (17.5%) reported using educational materials such as books and brochures, indicating that traditional educational resources are less frequently utilized compared to digital means.

Information from friends/peers is a source for 180 participants (30.0%), suggesting that peer communication remains important but less relied upon. Family members are cited by 155 women (25.8%) as a source of STI information, reflecting the role of familial discussions in health education, although this is less predominant compared to other sources.

Table 5 highlights significant regional differences in STI knowledge among women in Saudi Arabia. The Eastern region reports the highest average knowledge score of 78.9 (±10.7), suggesting relatively higher awareness and understanding of STIs. In comparison, the Southern region exhibits the lowest average score at 65.7 (±14.1), indicating considerable gaps in STI knowledge. The Central region, with an average score of 75.3 (±12.4), and the Western region, scoring 72.4 (±13.2), show moderate levels of awareness. Meanwhile, the Northern region has a lower average score of 68.5 (±15.3), reflecting substantial variability in knowledge levels within this area.

Table 6 presents the impact of various demographic variables on the level of STI knowledge among women in Saudi Arabia, using Chi-square analysis. The analysis reveals that age, educational level, and marital status significantly influence STI knowledge, while residential area does not show a significant effect. Specifically, age demonstrates a significant variation (χ^2^ = 12.54, df = 4, *p* = 0.014), indicating that younger and older age groups differ in their knowledge levels, potentially reflecting generational differences in education or exposure to information. Educational level shows the most substantial impact (χ^2^ = 28.79, df = 7, *p* < 0.001), with higher education correlating with better STI knowledge, underscoring the critical role of formal education in disseminating sexual health information. Marital status also significantly affects STI knowledge (χ^2^ = 9.33, df = 3, *p* = 0.025), suggesting that marital experiences may influence awareness and understanding of STIs. Conversely, residential area does not significantly impact STI knowledge (χ^2^ = 6.42, df = 4, *p* = 0.169), indicating similar levels of knowledge across different regions.

The logistic regression analysis (Table 7) reveals significant predictors for high STI knowledge among women in Saudi Arabia. Age positively influences STI knowledge, with a slight increase in awareness per year (OR: 1.05, 95% CI: 1.01–1.09, *p* = 0.014). Education level is a critical factor; higher educational attainment, particularly at the bachelor’s level and above, is strongly associated with better STI knowledge, with Ph.D. holders having the highest odds (OR: 2.50, 95% CI: 1.96–3.20, *p* < 0.001). Geographic location also impacts knowledge, with women from Northern and Southern regions displaying lower awareness compared to those from Central regions, although these differences are not always statistically significant. Marital status influences knowledge levels, with married women showing greater awareness (OR: 1.40, 95% CI: 1.10–1.79, *p* = 0.006). Sources of information, notably the internet and social media, significantly enhance STI knowledge compared to traditional sources like healthcare providers, highlighting the growing role of digital media in health education. Behavioral factors such as undergoing STI testing, discussing STIs with partners, using protection, and receiving HPV vaccination are strongly linked to higher STI knowledge.

## 4. Discussion

The present study provides a comprehensive assessment of women’s knowledge, awareness, and misconceptions regarding sexually transmitted infections (STIs) in Saudi Arabia. The findings reveal significant variations in STI knowledge across different demographic groups and highlight the need for targeted educational interventions to address existing gaps and misconceptions.

One of the key findings is the moderate overall level of STI knowledge among the participants. While a significant proportion (72.5%) could identify common STIs, there were notable knowledge deficits in understanding transmission routes (31.7% lacking knowledge), recognizing symptoms (40.8% lacking knowledge), and being aware of prevention methods (35.2% lacking knowledge). These findings are consistent with previous studies conducted in Saudi Arabia and other Middle Eastern countries, which have highlighted gaps in STI knowledge and awareness among the general population [27,41].

This study also uncovered several prevalent misconceptions about STIs among Saudi women. A substantial 38% of participants believed that STIs could be transmitted through casual contact, which contradicts the well-established understanding that STIs are primarily spread through sexual contact or exposure to infected bodily fluids [14,15]. This misconception may contribute to stigma and discrimination against individuals with STIs, as well as a lack of understanding about appropriate preventive measures.

Another concerning misconception was the belief held by 30% of respondents that only individuals with multiple sexual partners are at risk of contracting STIs. This misconception overlooks the fact that even individuals in monogamous relationships can acquire STIs through other means, such as vertical transmission or previous sexual encounters [42]. Additionally, 32% of participants believed that STIs are always visible with symptoms, which is inaccurate as many STIs can be asymptomatic, particularly in the early stages [43]. This misunderstanding could lead to delays in seeking treatment and contribute to the further spread of infections.

This study also revealed variations in STI knowledge across different demographic groups, highlighting the need for targeted educational interventions. Age was found to be a significant predictor of STI knowledge, with older participants generally demonstrating higher awareness. This finding aligns with previous research suggesting that life experiences and exposure to health information may contribute to increased knowledge about STIs among older individuals [44].

Education level emerged as a critical factor influencing STI knowledge, with higher educational attainment strongly associated with better awareness. This finding is consistent with numerous studies that have linked higher education levels to improved health literacy and understanding of complex health topics [45,46]. Individuals with advanced degrees, such as bachelor’s, master’s, and Ph.D., exhibited the highest levels of STI knowledge, underscoring the importance of incorporating comprehensive sexual health education into formal educational curricula.

Marital status also played a role in shaping STI knowledge, with married women displaying greater awareness compared to single individuals. This finding may be attributed to the increased likelihood of discussions about sexual health and STI prevention within marital relationships [47]. Additionally, married individuals may have greater exposure to healthcare services and counseling related to family planning and reproductive health, which could contribute to their enhanced knowledge about STIs [48].

The sources of information about STIs also influenced knowledge levels among the participants. The internet and social media emerged as significant predictors of higher STI knowledge, reflecting the growing influence of digital platforms in disseminating health information [49]. This finding aligns with global trends, where individuals, particularly younger populations, increasingly rely on online resources and social media for health-related information [50]. However, it is crucial to ensure that the information available on these platforms is accurate, reliable, and culturally appropriate.

Behavioral factors, such as undergoing STI testing, discussing STIs with partners, using protection during sexual encounters, and receiving the HPV vaccination, were strongly associated with higher levels of STI knowledge [51]. These findings suggest that individuals who actively engage in preventive behaviors and seek healthcare services related to sexual health are more likely to be well informed about STIs [52,53]. This underscores the importance of promoting positive health-seeking behaviors and ensuring access to comprehensive sexual and reproductive health services.

STI knowledge across different geographic regions was considered a study variable to identify potential disparities influenced by regional cultural, educational, and healthcare access factors. The hypothesis was that women’s STI knowledge might vary significantly by region due to differences in these factors. For instance, urban areas might have better access to healthcare services and educational resources, leading to higher levels of STI awareness, whereas rural areas might face barriers such as limited healthcare facilities and lower levels of education.

The differences in STI knowledge across regions can be attributed to several factors. Urban areas typically have more healthcare facilities, public health campaigns, and educational institutions that promote awareness about STIs. In contrast, rural regions may have fewer resources, less access to healthcare services, and cultural barriers that prevent open discussions about sexual health. Additionally, regional variations in socioeconomic status, literacy rates, and the presence of public health interventions could also contribute to these differences.

Addressing these regional disparities is crucial for developing targeted educational interventions and public health strategies. By understanding and addressing the specific needs and challenges of each region, we can enhance STI awareness and prevention efforts, ultimately improving public health outcomes across Saudi Arabia.

Countries like the Netherlands and Sweden have implemented comprehensive sex education programs that are age-appropriate and culturally sensitive, providing continuous education from an early age through adolescence [28]. Public health campaigns in the United States and Australia use mass media to disseminate information about STIs, engaging the public and reducing stigma [54]. Training healthcare providers to discuss sexual health issues openly and sensitively, as carried out in the UK, helps normalize conversations and encourages individuals to seek information and care. Engaging community leaders and influencers, as practiced in some African countries, effectively promotes STI awareness tailored to cultural contexts. Utilizing digital platforms and mobile health (mHealth) initiatives, seen in Canada and the United States, offers discreet and accessible information about STIs, encouraging regular testing and connecting users with healthcare services [55]. Adapting these strategies to the Saudi context, incorporating cultural and religious considerations, can significantly improve STI awareness and prevention efforts among women in Saudi Arabia.

### 4.1. Implications of the Study

The findings of this study have significant implications for public health policies and interventions aimed at improving STI awareness and prevention in Saudi Arabia. Firstly, the identification of knowledge gaps and misconceptions emphasizes the need for comprehensive and culturally tailored educational campaigns and programs. These initiatives should leverage various communication channels, including social media and digital platforms, to effectively reach diverse demographic groups.

Secondly, this study underscores the importance of integrating comprehensive sexual health education into formal educational curricula at different levels. This approach ensures that accurate and age-appropriate information about STIs is disseminated to students, fostering a well-informed and knowledgeable population.

Furthermore, this study highlights the crucial role of healthcare providers in enhancing STI awareness. Providing training to healthcare professionals to discuss sexual health issues in a culturally sensitive manner can enhance their capacity to offer effective counseling and education to patients.

Community engagement and partnerships with religious and cultural leaders are also vital implications arising from this study. By addressing cultural barriers and leveraging the influence of community leaders, educational initiatives can gain greater acceptance and reach a broader audience, promoting open discussions about STIs and reducing associated stigma.

Additionally, the findings suggest the need to improve access to STI testing and treatment services, particularly in underserved regions. Telemedicine and mobile health (mHealth) initiatives can play a significant role in expanding access to sexual and reproductive health services, especially in remote or resource-limited areas.

### 4.2. Limitations of the Study

This study has several limitations. First, the term ‘illiterate’ was inaccurately used as all participants had basic reading skills necessary to complete the questionnaire. Future research should include alternative methods such as verbal surveys for truly illiterate individuals. Second, recruitment through social media and email may have introduced selection bias, excluding those less engaged with digital platforms. No monetary incentives were provided, which could have further limited participation. Third, the study assessed knowledge of ‘common STIs’ (chlamydia, gonorrhea, syphilis, HPV, herpes) but did not differentiate between individual STIs and overall STI knowledge, which future studies should address. Fourth, inconsistencies in reporting statistical significance across regions suggest flaws in our analysis. The hypothesis aimed to identify disparities influenced by regional factors, requiring further investigation. Fifth, self-reported data may introduce social desirability bias, affecting accuracy. Lastly, the cross-sectional design provides a snapshot in time without capturing changes or causality. Longitudinal studies are needed to evaluate the effectiveness of educational interventions. Addressing these limitations will help develop more inclusive and accurate strategies to improve STI awareness and prevention among women in Saudi Arabia.

## 5. Conclusions

This comprehensive study provides valuable insights into the current state of STI awareness and knowledge among women in Saudi Arabia. The findings highlight the need for concerted efforts to enhance STI education and address the identified knowledge gaps, misconceptions, and demographic variations.

By implementing targeted interventions, such as culturally tailored educational campaigns, integration of sexual health education into formal curricula, training for healthcare providers, and community engagement with religious and cultural leaders, Saudi Arabia can promote a better understanding of STIs, their transmission, symptoms, and prevention strategies.

Collaboration between government agencies, healthcare providers, educational institutions, and community leaders is essential to create a supportive environment for open discussions about sexual health and to implement culturally appropriate educational initiatives. These collective efforts will contribute to improved sexual and reproductive health outcomes, reduce the burden of STIs, and foster a more informed and healthy society.

Additionally, leveraging digital platforms and mobile health initiatives can enhance the reach and accessibility of STI education and healthcare services, particularly in remote or resource-limited areas. Continuous research and evaluation of educational interventions will be crucial to track progress and refine strategies for maximum effectiveness.

Ultimately, this study serves as a catalyst for addressing the critical issue of STI awareness and prevention in Saudi Arabia, paving the way for a future where women are empowered with accurate knowledge, free from stigma, and equipped to make informed decisions about their sexual and reproductive health.

## Figures and Tables

**Table 1:** Demographic characteristics of study participants.

Demographic Variable	Categories	Frequency (n)	Percentage (%)
Age group	18–23	117	19.5
	24–30	152	25.3
	31–40	141	23.5
	41–50	109	18.2
	51–55	81	13.5
Educational level	Non-formal Education	28	4.7
	Elementary school	41	6.8
	Intermediate school	68	11.3
	Secondary school	103	17.2
	Diploma	87	14.5
	Bachelor’s degree	188	31.3
	Master’s degree	52	8.7
	Ph.D.	33	5.5
Residential area	Central	132	22.0
	Eastern	111	18.5
	Northern	88	14.7
	Southern	118	19.7
	Western	151	25.2
Marital status	Single	209	34.8
	Married	278	46.3
	Divorced	73	12.2
	Widowed	40	6.7

**Table 2:** Overall knowledge of STIs among participants.

Knowledge Question	Correct Response Rate (%)	Incorrect Response Rate (%)
Identified common STIs	72.5% (435)	27.5% (165)
Understanding of STI transmission routes	68.3% (410)	31.7% (190)
Knowledge of STI symptoms	59.2% (355)	40.8% (245)
Awareness of STI prevention methods	64.8% (389)	35.2% (211)

**Table 3:** Misconceptions about STIs.

Misconception Statement	Agree (%)	Disagree (%)	Not Sure (%)
STIs can be transmitted through casual contact	228 (38%)	294 (49%)	78 (13%)
Only people with multiple sexual partners get STIs	180 (30%)	336 (56%)	84 (14%)
STIs are always visible with symptoms	192 (32%)	312 (52%)	96 (16%)
Condoms completely eliminate the risk of STIs	240 (40%)	288 (48%)	72 (12%)

**Table 4:** Sources of information on STIs.

Information Source	Frequency (n)	Percentage (%)
Healthcare providers	215	35.8
Educational materials (books, brochures)	105	17.5
Internet/websites	260	43.3
Social media	245	40.8
Friends/peers	180	30.0
Family members	155	25.8

**Table 5:** Regional differences in STI knowledge.

Residential Area	Average Knowledge Score (Mean ± SD)
Central	75.3 ± 12.4
Eastern	78.9 ± 10.7
Northern	68.5 ± 15.3
Southern	65.7 ± 14.1
Western	72.4 ± 13.2

**Table 6:** Impact of demographic variables on STI knowledge (Chi-square analysis).

Demographic Variable	Chi-Square Value	Degrees of Freedom (df)	*p*-Value
Age group	12.54	4	0.014
Educational level	28.79	7	<0.001
Residential area	6.42	4	0.169
Marital status	9.33	3	0.025

**Table 7:** Logistic regression analysis of predictors for high STI knowledge.

Predictor Variable	Categories	Odds Ratio (OR)	95% Confidence Interval (CI)	*p*-Value
Age	Per-year increase	1.05	1.01–1.09	0.014
Education level	Non-formal Education (reference)	-	-	-
	Elementary school	1.20	0.92–1.58	0.150
	Intermediate school	1.45	1.12–1.89	0.007
	Secondary school	1.70	1.32–2.18	<0.001
	Diploma	1.85	1.44–2.38	<0.001
	Bachelor’s degree	2.10	1.66–2.65	<0.001
	Master’s degree	2.25	1.78–2.85	<0.001
	Ph.D.	2.50	1.96–3.20	<0.001
Residential area	Central (reference)	-	-	-
	Eastern	0.92	0.70–1.20	0.523
	Northern	0.75	0.56–1.01	0.061
	Southern	0.80	0.60–1.06	0.126
	Western	1.05	0.80–1.38	0.700
Marital status	Single (reference)	-	-	-
	Married	1.40	1.10–1.79	0.006
	Divorced	1.30	0.92–1.83	0.135
	Widowed	1.10	0.73–1.65	0.648
Information source	Healthcare providers (reference)	-	-	-
	Educational materials	1.30	1.02–1.66	0.035
	Internet/websites	1.40	1.11–1.76	0.004
	Social media	1.35	1.05–1.74	0.021
	Friends/peers	1.15	0.78–1.68	0.479
	Family members	1.10	0.71–1.70	0.665
STI testing	Never tested (reference)	-	-	-
	Tested	1.55	1.23–1.95	<0.001
Discussing STIs	Never discussed (reference)	-	-	-
	Discussed	1.70	1.35–2.13	<0.001
Protection use	Never used protection (reference)	-	-	-
	Used protection	1.65	1.30–2.10	<0.001
HPV vaccination	Not vaccinated (reference)	-	-	-
	Vaccinated	1.60	1.24–2.06	<0.001

## Data Availability

The data supporting the reported results are available on request from the corresponding author. The data are not publicly available due to privacy and ethical restrictions.

## References

[1] Otu A., Danhoundo G., Toskin I., Govender V., Yaya S. (2021). Refocusing on Sexually Transmitted Infections (STIs) to Improve Reproductive Health: A Call to Further Action. Reprod. Health.

[2] Van Gerwen O.T., Muzny C.A., Marrazzo J.M. (2022). Sexually Transmitted Infections and Female Reproductive Health. Nat. Microbiol..

[3] Parker J.N., Choi S.K., Bauermeister J.A., Bonar E.E., Carrico A.W., Stephenson R. (2022). HIV and Sexually Transmitted Infection Testing among Substance-Using Sexual and Gender Minority Adolescents and Young Adults: Baseline Survey of a Randomized Controlled Trial. JMIR Public Health Surveill..

[4] Zaki S.A., Naous J., Ghanem A., Abou Abbas D., Tomb R., Ghosn J., Assi A. (2021). Prevalence of STIs, Sexual Practices and Substance Use among 2083 Sexually Active Unmarried Women in Lebanon. Sci. Rep..

[5] Aseeri I.A., AlOtaibi M.N., Alzahrani W.J., Althomali M.A., Alattar H.A., Althobity A.F. (2023). Public Awareness About Sexually Transmitted Diseases in Taif, Saudi Arabia. Cureus.

[6] Alomair N., Alageel S., Davies N., Bailey J.V. (2023). Muslim Women’s Knowledge, Views, and Attitudes towards Sexually Transmitted Infections in Saudi Arabia: A Qualitative Study. PLoS ONE.

[7] ALDosari M., Abuzied T., Alhussaini N., Althobaiti M., Tumihi Y., Alhumaid T., AlRumayan A. (2024). The Influence of Gender and Education on Awareness about Sexually Transmitted Diseases among King Saud University and Imam Mohammed Ibn Saud Islamic University Students in Riyadh, Saudi Arabia. Cureus.

[8] Madani T.A. (2006). Sexually Transmitted Infections in Saudi Arabia. BMC Infect. Dis..

[9] Ford J.V., Barnes R., Rompalo A., Hook E.W. (2013). Sexual Health Training and Education in the U.S. Public Health Rep..

[10] Chavula M.P., Zulu J.M., Hurtig A.-K. (2022). Factors Influencing the Integration of Comprehensive Sexuality Education into Educational Systems in Low- and Middle-Income Countries: A Systematic Review. Reprod. Health.

[11] Subbarao N.T., Akhilesh A. (2017). Knowledge and Attitude about Sexually Transmitted Infections Other than HIV among College Students. Indian J. Sex. Transm. Dis. AIDS.

[12] Alomair N., Alageel S., Davies N., Bailey J.V. (2023). Muslim Women’s Perspectives on the Barriers to Sexually Transmitted Infections Testing and Diagnosis in Saudi Arabia. Front. Public Health.

[13] Alomair N., Alageel S., Davies N., Bailey J.V. (2023). Muslim Women’s Views and Experiences of Family Planning in Saudi Arabia: A Qualitative Study. BMC Womens. Health.

[14] Malli I.A., Kabli B.A., Alhakami L.A. (2023). Sexually Transmitted Diseases among Saudi Women: Knowledge and Misconceptions. Int. J. Environ. Res. Public Health.

[15] Balbeesi A., Mohizea S. (2017). Knowledge and Misconceptions of Saudi Women about Sexually Transmitted Infections. J. Egypt. Public Health Assoc..

[16] Fageeh W., Fakieh B., Addas M., Baghdadi R., Almokri R., Sait S., Fagih S., Alahmadi S. (2022). Knowledge and Attitudes towards Sexually Transmitted Illnesses (STIs) among the General Population of Saudi Arabia. Clin. Exp. Obstet. Gynecol..

[17] Zajacova A., Lawrence E.M. (2018). The Relationship Between Education and Health: Reducing Disparities through a Contextual Approach. Annu. Rev. Public Health.

[18] Huang R., Yang Y., Zajacova A., Zimmer Z., Li Y., Grol-Prokopczyk H. (2023). Educational Disparities in Joint Pain within and across US States: Do Macro Sociopolitical Contexts Matter?. Pain.

[19] Alomair N., Alageel S., Davies N., Bailey J.V. (2022). Sexual and Reproductive Health Knowledge, Perceptions and Experiences of Women in Saudi Arabia: A Qualitative Study. Ethn. Health.

[20] Alquaiz A.M., Almuneef M.A., Minhas H.R. (2012). Knowledge, Attitudes, and Resources of Sex Education among Female Adolescents in Public and Private Schools in Central Saudi Arabia. Saudi Med. J..

[21] Chemaitelly H., Finan R.R., Racoubian E., Aimagambetova G., Almawi W.Y. (2024). Estimates of the Incidence, Prevalence, and Factors Associated with Common Sexually Transmitted Infections among Lebanese Women. PLoS ONE.

[22] Alfaqeeh G., Cook E.J., Randhawa G., Ali N. (2017). Access and Utilisation of Primary Health Care Services Comparing Urban and Rural Areas of Riyadh Providence, Kingdom of Saudi Arabia. BMC Health Serv. Res..

[23] Habib S.S., Jamal W.Z., Zaidi S.M.A., Siddiqui J.-U.-R., Khan H.M., Creswell J., Batra S., Versfeld A. (2021). Barriers to Access of Healthcare Services for Rural Women—Applying Gender Lens on TB in a Rural District of Sindh, Pakistan. Int. J. Environ. Res. Public Health.

[24] El-Tholoth H., Alqahtani F., Aljabri A., Alfaryan K., Alharbi F., Alhowaimil A., Alkharji A., Alrwaily A., Obied A., Al-Afraa T. (2018). Knowledge and Attitude about Sexually Transmitted Diseases among Youth in Saudi Arabia. Urol. Ann..

[25] Gabarron E., Wynn R. (2016). Use of Social Media for Sexual Health Promotion: A Scoping Review. Glob. Health Action.

[26] Yang N., Wu D., Zhou Y., Huang S., He X., Tucker J., Li X., Smith K.M., Jiang X., Wang Y. (2021). Sexual Health Influencer Distribution of HIV/Syphilis Self-Tests Among Men Who Have Sex with Men in China: Secondary Analysis to Inform Community-Based Interventions. J. Med. Internet Res..

[27] Albanghali M.A., Othman B.A. (2020). A Cross-Sectional Study on the Knowledge of Sexually Transmitted Diseases among Young Adults Living in Albaha, Saudi Arabia. Int. J. Environ. Res. Public Health.

[28] Leung H., Shek D., Leung E., Shek E. (2019). Development of Contextually-Relevant Sexuality Education: Lessons from a Comprehensive Review of Adolescent Sexuality Education across Cultures. Int. J. Environ. Res. Public Health.

[29] Ramchandani M.S., Golden M.R. (2019). Confronting Rising STIs in the Era of PrEP and Treatment as Prevention. Curr. HIV/AIDS Rep..

[30] St. Lawrence J.S., Fortenberry J.D. (2007). Behavioral Interventions for STDs: Theoretical Models and Intervention Methods. Behavioral Interventions for Prevention and Control of Sexually Transmitted Diseases.

[31] Kumar R., Verma A., Shome A., Sinha R., Sinha S., Jha P.K., Kumar R., Kumar P., Shubham, Das S. (2021). Impacts of Plastic Pollution on Ecosystem Services, Sustainable Development Goals, and Need to Focus on Circular Economy and Policy Interventions. Sustainability.

[32] Alghamdi S.M., Alsulayyim A.S., Alqahtani J.S., Aldhahir A.M. (2021). Digital Health Platforms in Saudi Arabia: Determinants from the COVID-19 Pandemic Experience. Healthcare.

[33] Altameem T. (2011). Contextual Mobile Learning System for Saudi Arabian Universities. Int. J. Comput. Appl..

[34] Muessig K.E., Pike E.C., LeGrand S., Hightow-Weidman L.B. (2013). Mobile Phone Applications for the Care and Prevention of HIV and Other Sexually Transmitted Diseases: A Review. J. Med. Internet Res..

[35] Martin P., Alberti C., Gottot S., Bourmaud A., de La Rochebrochard E. (2023). Young People’s Proposals for a Web-Based Intervention for Sexual Health Promotion: A French Qualitative Study. BMC Public Health.

[36] Bearinger L.H., Sieving R.E., Ferguson J., Sharma V. (2007). Global Perspectives on the Sexual and Reproductive Health of Adolescents: Patterns, Prevention, and Potential. Lancet.

[37] Garcia P.J., Miranda A.E., Gupta S., Garland S.M., Escobar M.E., Fortenberry J.D. (2021). The Role of Sexually Transmitted Infections (STI) Prevention and Control Programs in Reducing Gender, Sexual and STI-Related Stigma. EClinicalMedicine.

[38] Rajabi M., Ebrahimi P., Aryankhesal A. (2021). Collaboration between the Government and Nongovernmental Organizations in Providing Health-Care Services: A Systematic Review of Challenges. J. Educ. Health Promot..

[39] Mumtaz H., Riaz M.H., Wajid H., Saqib M., Zeeshan M.H., Khan S.E., Chauhan Y.R., Sohail H., Vohra L.I. (2023). Current Challenges and Potential Solutions to the Use of Digital Health Technologies in Evidence Generation: A Narrative Review. Front. Digit. Health.

[40] AlNujaidi H., AlSaif A., Saleem ALAnsary N., Althumiri N., BinDhim N. (2023). The Knowledge and Determinants of Sexual Health and Sexual Transmitted Infections among Women in Saudi Arabia: A Nationwide Survey. Int. J. Women’s Health.

[41] Alshemeili A., Alhammadi A., Alhammadi A., Al Ali M., Alameeri E.S., Abdullahi A.S., Abu-Hamada B., Sheek-Hussein M.M., Al-Rifai R.H., Elbarazi I. (2023). Sexually Transmitted Diseases Knowledge Assessment and Associated Factors among University Students in the United Arab Emirates: A Cross-Sectional Study. Front. Public Health.

[42] Llata E., Cuffe K.M., Picchetti V., Braxton J.R., Torrone E.A. (2021). Demographic, Behavioral, and Clinical Characteristics of Persons Seeking Care at Sexually Transmitted Disease Clinics—14 Sites, STD Surveillance Network, United States, 2010–2018. MMWR Surveill. Summ..

[43] Mansor N., Ahmad N., Rahman H.A. (2020). Determinants of Knowledge on Sexually Transmitted Infections among Students in Public Higher Education Institutions in Melaka State, Malaysia. PLoS ONE.

[44] Smith M.L., Bergeron C.D., Goltz H.H., Coffey T., Boolani A. (2020). Sexually Transmitted Infection Knowledge among Older Adults: Psychometrics and Test–Retest Reliability. Int. J. Environ. Res. Public Health.

[45] Annang L., Walsemann K.M., Maitra D., Kerr J.C. (2010). Does Education Matter? Examining Racial Differences in the Association between Education and STI Diagnosis among Black and White Young Adult Females in the U.S. Public Health Rep..

[46] Sleiman V., Obeid S., Sacre H., Salameh P., Hallit S., Hallit R. (2023). Knowledge, Attitudes, and Practices of Lebanese University Students Related to Sexually Transmitted Diseases: A Cross-Sectional Study. Croat. Med. J..

[47] Liu Y., Li D., Vermund S.H., Zhang C., Ruan Y., Yin L., Liu H., Amico K.R., Shao Y., Qian H.-Z. (2016). Associations of Current Marital Status and Living Arrangements with HIV and Syphilis Risk: Findings from a Community-Based Sample of Men Who Have Sex with Men in China. AIDS Care.

[48] Kaur W., Balakrishnan V., Zhi Wei I.N., Chen A.Y.Y., Ni Z. (2023). Understanding Women’s Knowledge, Awareness, and Perceptions of STIs/STDs in Asia: A Scoping Review. Healthcare.

[49] Mohamad Shakir S.M., Wong L.P., Abdullah K.L., Adam P. (2019). Factors Associated with Online Sexually Transmissible Infection Information Seeking among Young People in Malaysia: An Observational Study. Sex. Health.

[50] Freeman J.L., Caldwell P.H.Y., Scott K.M. (2023). How Adolescents Trust Health Information on Social Media: A Systematic Review. Acad. Pediatr..

[51] Mullins T.L.K., Zimet G.D., Rosenthal S.L., Morrow C., Ding L., Huang B., Kahn J.A. (2016). Human Papillomavirus Vaccine-Related Risk Perceptions and Subsequent Sexual Behaviors and Sexually Transmitted Infections among Vaccinated Adolescent Women. Vaccine.

[52] Meyer-Weitz A., Reddy P., Van den Borne H.W., Kok G., Pietersen J. (2000). Health Care Seeking Behaviour of Patients with Sexually Transmitted Diseases: Determinants of Delay Behaviour. Patient Educ. Couns..

[53] Shimie A.W., Gashu K.D., Shiferaw A.M., Mengiste S.A. (2022). Information-Seeking Behavior on Sexually Transmitted Infections and Its Associated Factors among University Students in Ethiopia: A Cross-Sectional Study. Reprod. Health.

[54] Riddell J., Cleary A., Dean J.A., Flowers P., Heard E., Inch Z., Mutch A., Fitzgerald L., McDaid L. (2024). Social Marketing and Mass Media Interventions to Increase Sexually Transmissible Infections (STIs) Testing among Young People: Social Marketing and Visual Design Component Analysis. BMC Public Health.

[55] Venetis M.K., Meyerson B.E., Friley L.B., Gillespie A., Ohmit A., Shields C.G. (2017). Characterizing Sexual Orientation Disclosure to Health Care Providers: Lesbian, Gay, and Bisexual Perspectives. Health Commun..

